# Oral changes in cocaine abusers: an integrative review

**DOI:** 10.1016/j.bjorl.2021.04.011

**Published:** 2021-05-14

**Authors:** César Antonio Araújo Melo, Hanna Rabech Garcia Guimarães, Raphael Crhistian Fernandes Medeiros, Georgia Costa de Araújo Souza, Patrícia Bittencourt Dutra dos Santos, Ana Clara Soares Paiva Tôrres

**Affiliations:** Universidade do Estado do Rio Grande do Norte (UERN), Campus Avançado de Caicó, Departamento de Odontologia, Caicó, RN, Brazil

**Keywords:** Cocaine-related disorders, Oral manifestations, Diagnosis, Therapeutics, Dentistry

## Abstract

**Introduction:**

Cocaine is one of the most often used psychoactive drugs worldwide, being extracted from the leaves of *Erytroxylus coca* plant. Its abusive use can trigger several consequences for the human body, including the oral cavity.

**Objective:**

To identify the oral disorders that are most commonly found in individuals who abuse cocaine, in addition to the main diagnostic and treatment methods.

**Methods:**

An integrative review was carried out on the databases: LILACS, BBO, LIS, MEDLINE, SciELO, Science Direct and PubMed. The following keywords were used: “*Cocaína*”, “*Boca*”, “*Palato*” and “*Odontologia*”, together with their synonyms and variations in English, obtained from DeCS and MeSH. The inclusion criteria were original articles, articles in Portuguese, English and Spanish, studies involving individuals, without restriction related to the year of publication. Animal studies, literature reviews, book chapters, theses and dissertations were excluded.

**Results:**

In total, 1373 records were identified. Of these, 22 articles were selected to comprise the review. Several oral alterations caused by cocaine abuse were found, primarily perforation of the palate, predisposition to periodontal diseases, temporomandibular disorders, bruxism, damage to oral tissues, dental caries, destructive lesions of the facial midline, xerostomia and ageusia. Among the diagnostic methods used by the professionals, anamnesis, intraoral examinations and head and neck computed tomography were the most frequently mentioned. As for treatment, in patients with palatal perforation, the reconstruction of the affected area or the use of prosthetic obturators is carried out.

**Conclusion:**

The management of these patients is not an easy task, as many of the users do not even seek professional help. The health professionals must be able to recognize these manifestations and alterations to establish timely and accurate diagnosis and treatment planning.

## Highlights

• Integrative review aiming to detect oral changes in cocaine abusers.
• The main oral changes, methods of diagnosis and treatment were identified.
• Good anamnesis and qualified health professionals are necessary.

## Introduction

A psychoactive drug is defined as a chemical, natural or synthetic product that, when administered by any route (inhalation, ingestion, intramuscular, intravenous) acts on the central nervous system and triggers physical and/or psychiatric alterations, causing changes in sensations or modifying the psychological state, i.e., altering the individual’s behavior.^1^

The consumption of psychoactive substances can be influenced by the users’ social context and factors such as family income, level of schooling and place of residence.^2^ In this sense, drug abuse is considered a public health problem due to the systemic and behavioral consequences.^3^, ^4^

Among the most common and most frequently used illicit drugs worldwide is mainly *Cannabis sativa*, popularly known as marijuana.^5^, ^6^ In addition, there are amphetamines, ecstasy, opiates and cocaine.^7^ Benzoylmethylecgonine is a relatively recent drug among the psychoactive substances used by humans over time and can be found in and extracted from the leaves of the coca plant (*Erytroxylus coca*), which can reach the consumer through three different forms: in the form of salt, cocaine hydrochloride, and as powdered cocaine, which can be aspirated or dissolved in water for intravenous use.^8^

Derived from cocaine, crack cocaine consists of a base, transformed into crystals, poorly soluble in water and volatile when heated, which can be smoked in pipes. Regardless of the type of drug use, all the effects are caused by the use of both; however, when smoked, they show greater potency.^8^

Currently, cocaine is classified as a psychoactive substance that belongs to stimulating drugs that alter brain function, making it more active, acting on the central nervous system.^9^, ^10^ The consumption of this drug can be carried out in several ways; one of the main forms of cocaine administration is the intranasal route.^11^ A few minutes after inhalation, a feeling of euphoria occurs, which lasts around 20−90 min.

Moreover, cocaine users can rub the drug on gingival tissue due to the similar architecture of the nasal and oral mucosa and abundant vascularization.^12^ However, with this type of use, when rubbed on the gingival surface for more effective absorption, powdered cocaine can lead to irritation of this mucosa. *E. coca* absorption by the mucosa can cause oral lesions as a result of decreased blood supply due to vasoconstriction in the affected region, resulting in tissue necrosis.^13^

As a consequence, drug abuse can cause or result in the occurrence of physical problems such as cardiac complications, respiratory depression, liver cirrhosis, nephropathy, or it can indirectly cause infectious diseases, such as hepatitis, AIDS and tuberculosis. It can also cause disability and mental disorders, such as depression. These conditions can progress to more advanced stages and cause significant disorders, as it often takes addicted patients some time until they seek medical care and they do so when symptoms worsen.^14^

In addition to the consequences of cocaine use for one’s general health and the systemic effects of the drug, it is also necessary to consider the occurrence of oral changes in users,^15^ since the substance use can directly affect the dental tissues and the oral mucosa, which may cause xerostomia, changes in salivary flow, enamel erosion and abrasion, atypical caries, tooth loss^14^ and gingival lesions.^10^ Moreover, the regular use of cocaine can have serious orofacial effects, such as perforation of the nasal septum and palate, gingival lesions and tooth surface erosion, in addition to being associated with changes in the sense of smell and chronic sinusitis.^16^ In this sense, the present study aimed to identify the oral alterations most commonly found in individuals who abuse cocaine, in addition to their diagnoses and treatments, available in scientific publications.

## Methods

The steps of this integrative literature review were independently carried out by three researchers following the recommendations of the Preferred Reporting Items for Systematic Reviews and Meta-Analyzes (PRISMA).^17^

For a better construction of the present study, the PICO strategy (population, intervention, comparison and outcome) was used to set up the question.^18^ Based on this strategy, the following question was created: What are the most common lesions found in the oral cavity in users who abuse cocaine?

### Search strategy

The search for the studies was carried out from September to November 2020, in the following databases: LILACS, BBO, LIS and MEDLINE via the Virtual Health Library (VHL) portal, Scientific Electronic Library Online (SciELO), Science Direct and MEDLINE via PubMed. Variable combinations of descriptors obtained from DeCS (Health Sciences Descriptors) and MeSH (Medical Subject Headings) in English and Portuguese (Table 1) were used in the search.

**Table 1:** Search strategy used in each database.

Portals/databases	Search strategy
LILACS, BBO, LIS e MEDLINE (BVS)	(“Cocaína” OR “Cocaine” OR “Transtornos Relacionados ao Uso de Cocaína” OR “Cocaine-Related Disorders” AND “Odontólogos” OR “Dentists” OR “Odontologia” OR “Dentistry” AND “Palato” OR “Palate” OR “Boca” OR “Mouth” OR “Maxila” OR “Maxilla”)
SciELO	(“*Cocaína” OR “Cocaine” AND “Palato” OR “Palate” OR “Boca” OR “Mouth” OR “Maxila” OR “Maxilla”)
Science Direct	(“Cocaína” OR “Cocaine” OR “Transtornos Relacionados ao Uso de Cocaína” OR “Cocaine-Related Disorders” AND “Odontologia” OR “Dentistry” AND “Palate” OR “Palato”)
PubMed	(“Cocaine” [MeSH Terms] OR “Cocaine-Related Disorders” [MeSH Terms] AND “Dentistry” [MeSH Terms] AND “Mouth” [MeSH Terms])

LILACS, *Literatura Latino-Americana e do Caribe em Ciências da Saúde*; BBO, *Bibliografia Brasileira de Odontologia*; LIS, *Localizador de Informação em Saúde*; BVS, *Biblioteca Virtual de Saúde*; SciELO, Scientific Electronic Library Online.

### Study eligibility criteria

Studies that showed results related to lesions found in the oral cavity of cocaine abusers were included. The inclusion criteria were: original articles, articles in Portuguese, English and Spanish, case reports, cross-sectional studies, experimental studies, observational studies and field studies. There were no restrictions regarding the year of publication. Studies in animals, literature reviews, book chapters, theses and dissertations were excluded.

### Study selection

After searching the databases, the titles and abstracts were listed in a standardized manner. Then, articles in duplicate were excluded, and based on the inclusion and exclusion criteria, the initial selection of studies that had the potential for full-text reading was performed. In case of disagreement, a fourth reviewer was consulted, and the decision was made by consensus. The full texts that were not available in the databases were requested directly from their authors. A manual search was also carried out in the list of references of the articles considered eligible. After reading the texts in full and deciding to include the articles in the present study, the most relevant results were extracted for sequential analysis.

## Results

**Figure 1:**
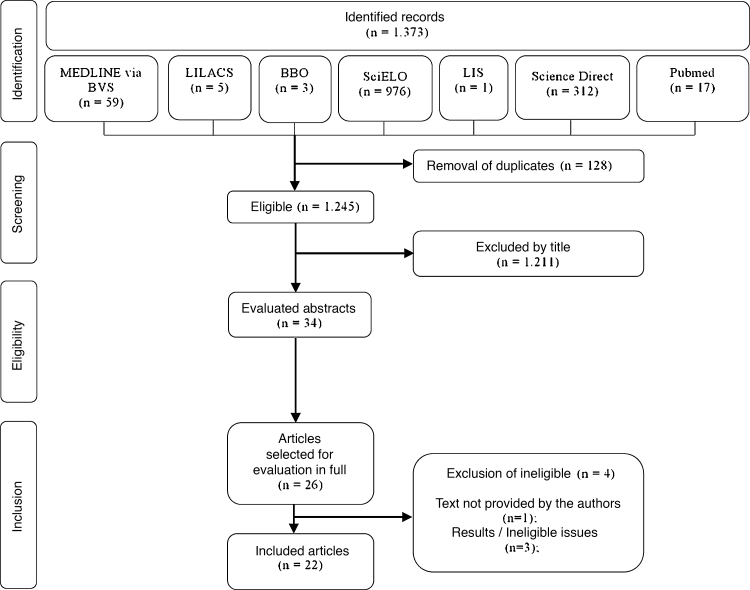
Flowchart with the search and screening strategy of the studies selected to constitute the review of oral alterations in cocaine users. 2020.

The electronic and manual search resulted in the identification of 1373 articles. Of these, after the initial exclusion by titles and abstracts, a total of 26 articles were selected according to the eligibility criteria. Finally, after reading the texts in full, 22 articles were included in the review. The study flowchart can be seen in Fig. 1.

The overall characteristics of the 22 selected articles can be seen in Table 2. The number of individuals in the studies ranged from at least one participant (case reports) to a maximum of 212 participants. The mean age of the individuals who participated in the studies ranged from 13 to a maximum of 70 years.

**Table 2:** General aspects of the selected articles in 2020.

Authors/year	Country	N. of patients	Age (years)	Gender	Design	Main oral lesions or alterations	Outcome
Antoniazzi et al. (2017)^19^	Brazil	80	–	–	Cross-sectional study	Reduction of salivary flow	Significant association between the use of crack-cocaine and reduced salivary flow.
Antoniazzi et al. (2018)^15^	Brazil	212	–	–	Cross-sectional study	Cell alterations in the oral mucosa (karyolysis, karyorrhexis, “broken egg” events and micronuclei)	Crack-cocaine users had a higher incidence of fundamental lesions.
Bains et al. (2005)^20^	United Kingdom	2	36 and 70	M	Case report and literature review	Palatal perforation	There is a relationship between palatal perforation and cocaine use.
Oñate et al. (2018)^21^	Chile	1	44	M	Case report	Destructive lesions in the midline	It was possible to diagnose a destructive lesion in the midline induced by cocaine, through the histological findings.
Blanco et al. (2017)^22^	Argentina	1	42	F	Case report	Palatal perforation	Palatal defects create functional difficulties related to speech and swallowing. Prosthetic rehabilitation with an obturator may be necessary to restore the function.
Brusati et al. (2009)^23^	Italy	1	46	F	Case report	Destruction of bone tissue in the facial midline, palate, anterior skull base, frontal bone and left orbital bone.	Extensive destruction of the craniofacial region, where four free flaps in association with the base of the skull and forehead are reconstituted with bone grafts and the use of implants.
Chaiben et al. (2014)^24^	Brazil	60	21 to 45	F	Experimental study	Loss of taste	The users found it difficult to recognize the salty, sweet and bitter tastes. Overall, the users mistook the salty flavor for sour or bitter taste and 20 (66.6%) were diagnosed with hypogeusia.
Chaparro- González et al. (2018)^25^	Venezuela	32	On average 37.7	M and F	Descriptive observational field study	Caries and periodontal disease	The most frequently used drugs were crack, followed by cocaine and marijuana. There is a predominance of caries (87.5%), followed by the presence of signs of periodontal disease.
Cury et al. (2018)^26^	Brazil	161	≥18	M	Cross-sectional observational study	Traumatic ulcer, actinic cheilitis, and fistula associated with retained tooth root	Prevalence of oral mucosa lesions (traumatic ulcer, actinic cheilitis, and fistula associated with retained tooth root) was higher in crack/cocaine addicts and crack/cocaine addiction was significantly associated with the occurrence of oral mucosa lesions.
Cosola et al. (2007)^27^	Italy and Spain	6	29 to 46	M and F	Case series	Palatal perforation	In all described cases, perforation of the nasal septum occurred first, followed by extensive destruction of the nasal and paranasal sinuses, with progression to palatal necrosis.
Dovigi et al. (2015)^28^	USA	1	41	M	Case Report	Palatal perforation	Midline destructive lesions induced by cocaine are a result of ischemic necrosis triggered by cocaine in a small subset of cocaine users, especially those who are predisposed to producing ANCA.
Hofstede et al. (2010)^29^	USA	1	48	M	Case Report	Midline destructive lesions	Palatal defects (erosion of the palate, nasal septum and inferior nasal turbinates) create functional difficulties related to speech and swallowing. Subsequent prosthetic rehabilitation with an obturator may be necessary to restore function in this group of patients.
Maia et al. (2012)^30^	Brazil	1	27	F	Case Report	Pyoderma Gangrenosum	The association between Pyoderma Gangrenosum and cocaine use is poorly described, since there are only two cases in the literature.
Martinez et al. (2014)^31^	Spain	1	45	F	Case Report and Literature Review	Destructive lesions of the midline with oronasal fistula	In the presence of necrotic lesions in the midline, with inconclusive nasal biopsies, the associated presence of palatal perforation is more typical of destructive midline lesions induced by cocaine or extranodal lymphoma than Wegener’s granulomatosis.
Rosas et al. (2006)^32^	Mexico	1	48	F	Case Report	Palatal perforation	The abusive intranasal cocaine use can induce necrosis and focal ischemia, which causes destruction of the secondary mucosa and the mid-facial bone.
Paradisi et al. (2020)^33^	Argentina	1	37	M	Case Report	Palatal perforation	The lesions present in the oral cavity as a result of medication use are irreversible if there is no control over time.
Pelo et al. (2008)^34^	Spain	1	45	M	Case Report	Oronasal communication	Le Fort I osteotomy and the use of the Bichat’s fat pad as a bilateral flap is an effective technique in the correction of small and medium-sized oronasal communications that cannot be resolved with a simple oral mucosa flap.
Candina et al. (2013)^35^	Cuba	43	13 to 29	M and F	Cross-sectional observational study	Periodontal diseases	Drug addicts had a high frequency of periodontal diseases, such as mild and moderate gingivitis and gingivitis with pocket formation, without periodontitis.
Shibli et al. (2005)^36^	Brazil	1	27	M	Case Report	Unusual onlay bone graft failure	Gingival recession and dental erosion have been associated with the local application of cocaine and its intense vasoconstrictor effect, which is responsible for these effects.
Sordi et al. (2017)^37^	Brazil	35	19 to 56	M and F	Cross-sectional study	Reduction in the salivary flow rate and mucosal lesions, aphthous stomatitis, frictional keratosis, candidiasis, tooth extraction scars and depapillation of the tongue	Illicit drug users, mainly of cocaine (77.15%), showed a reduction in the salivary flow rate and an increase in the number of lesions.
Stahelin et al. (2012)^38^	Brazil	1	43	F	Case Report	Midline destructive lesions	Although the ANCA test does not clearly differentiate the ANCA found in some patients with MDL from those in patients with WG, localized involvement and biopsy findings not typical of small vessel granulomatous vasculitis should be recognized as characteristics of cocaine-induced lesions.
Tsoukalas et al. (2000)^39^	USA	1	46	F	Case Report	Palatal perforation by three oronasal fistulas	Chronic nasal cocaine users can go to a dental office for routine care. If the dentist suspects cocaine abuse, all comprehensive treatment should be suspended until medical clearance.

ANCA, antineutrophil cytoplasmic antibodies; MDL, midline destructive lesions; M, male; F, female.

Regarding the type of study, of the 22 analyzed ones, 13 were case reports,^20^, ^21^, ^22^, ^23^, ^28^, ^29^, ^30^, ^31^, ^32^, ^33^, ^34^, ^36^, ^38^, ^39^ five were cross-sectional studies,^15^, ^19^, ^26^, ^35^^,^^37^ one was an experimental study,^24^ two were observational studies,^26^, ^35^ and one was a field study.^25^ Although literature reviews constituted an exclusion criterion, two studies^20^, ^31^ were included because they had more than one methodological type, being two case reports that contained literature reviews in their methodology.

Several oral alterations caused by cocaine abuse were identified in the studies, including palatal perforation,^20^, ^22^, ^26^, ^27^, ^28^, ^29^, ^31^, ^33^, ^34^ temporomandibular disorders (TMD),^25^, ^33^ bruxism,^25^, ^33^ predisposition to periodontal diseases, mainly gingivitis,^24^, ^25^, ^26^, ^35^ damage to oral tissues,^19^, ^24^ presence of caries,^24^, ^25^, ^26^, ^33^ destructive lesions of the facial midline,^21^, ^23^ xerostomia^19^, ^33^, ^39^ and ageusia.^24^

In most studies, cocaine use was associated with the use of other drugs. The isolated use of cocaine was described only in ten studies.^22^, ^23^, ^27^, ^29^, ^30^, ^31^, ^33^, ^34^, ^36^^,^^38^ There were also soft tissue alterations,^15^, ^37^ inflammatory responses,^15^, ^19^ and increased keratinization of the epithelium.^15^

Among the methods of diagnosis used by professionals to identify oral diseases, anamnesis,^31^, ^34^ intraoral examinations,^20^, ^22^, ^23^, ^25^^,^^29^, ^32^, ^33^, ^35^ head and neck computed tomography (CT),^21^, ^22^, ^23^, ^28^, ^31^, ^32^, ^33^ histopathological examinations^19^, ^32^, ^38^ and taste tests^24^ were the ones mentioned.

Regarding treatments, some authors stated that the patient’s oral manifestation must be taken into account and based on it, the best method of treatment that can be performed is determined. In patients with palatal perforation, reconstruction of the affected area is usually necessary, performed using flaps from the same palatal region or not,^20^, ^21^, ^23^, ^27^^,^^34^ or the use of adipose tissue from Bichat's fat pad^34^ or the use of prosthetic obturators,^20^, ^21^, ^27^, ^29^^,^^32^, ^33^ surgical reconstruction of the facial midline^23^ or even salivary stimulants.^19^

## Discussion

This integrative review addressed the most common oral disorders found in individuals who abuse cocaine. The studies included in the review were mostly case reports and cross-sectional studies. The results showed that the most frequent types of oral alterations were palatal perforation,^20^, ^22^, ^26^, ^27^, ^28^, ^29^, ^31^, ^33^, ^34^ bruxism,^25^, ^33^ periodontal diseases,^24^, ^25^, ^26^, ^35^ and presence of caries.^24^, ^25^, ^26^, ^33^ The main strategies used in the examination and diagnosis were intraoral examinations,^20^, ^22^, ^23^, ^25^^,^^29^, ^32^, ^33^, ^35^ and CT.^21^, ^22^, ^23^, ^28^, ^31^, ^32^, ^33^ Only a few authors mentioned strategies to rehabilitate or replace oral tissues partially or completely destroyed by cocaine use.^20^, ^21^, ^23^, ^27^^,^^34^

Regarding the most common oral manifestations, it is necessary to consider that because cocaine has a high vasoconstrictor effect, it is possible that its use leads to the ischemia of soft and hard tissues of the oral cavity and, consequently, their necrosis.^20^ This destructive process can cause ulcers or perforation in the hard and soft palate, culminating in an oronasal communication and a consequent nasal voice,^23^, ^25^, ^32^ difficulties in eating and drinking^20^, ^39^ and even nasal regurgitation.^20^, ^22^, ^27^, ^29^^,^^31^ These were the patients’ main motivations for seeking professional help. However, some studies mentioned that some users did not allow this identification, as they kept denying the drug use, aiming to avoid hospitalization or judgments.^20^, ^21^

Perforations can happen during the drug abuse period or even years after drug use discontinuation. For that to occur, the presence of an inflammatory process is necessary, through bacterial, viral^40^ or fungal infections, which are usually also associated with ageusia.^24^ In addition to these, inflammatory diseases associated with systemic diseases^21^, ^30^ or physical or chemical aggressions can be related to this type of alteration. In the case reported by Dovigi and Natarajan,^28^ an individual with extensive bone loss in the palate and nasal septum caused by years of cocaine use experienced an oronasal communication after burning the “palate”, making it impossible for him to eat comfortably, as everything invaded his nasal passage.

Regarding oronasal communications, the literature indicates that they can be small and transient,^20^, ^28^, ^31^ extensive^23^ or also multiple.^29^, ^33^, ^39^ As they are anatomically very close to the palatal region and because they lack cartilaginous vascularization, it is also possible they are commonly accompanied by nasal septum perforations and destruction.^22^, ^30^, ^31^, ^32^

In addition to the previously mentioned consequences, patients who are addicted to cocaine may also have muscle disorders and, therefore, possible temporomandibular disorder.^33^ Chaparro-González et al.^25^ corroborates this finding by identifying that users of drugs such as cocaine, methamphetamine and opioids suffer from bruxism, which results in a higher frequency of TMD. An evaluation of the presence of harmful oral habits in the analyzed sample shows that 59.4% had bruxism, and 37.5% had onychophagia.^25^

The form of cocaine use can be variable and the direct use of cocaine by rubbing it on the gums is one of them. This type of use acts as a substance purity test and can cause gingival recession, ulceration and necrosis.^20^, ^36^ An observational study showed that none of the addicts had periodontitis; however, 28 of the 43 patients had some form of gingival disease. Of the total number of affected individuals, five had mild gingivitis, 17 had moderate gingivitis and six had gingivitis with a periodontal pocket formation according to Russel’s index.^35^ Regarding oral manifestations in drug addicts, the most frequent condition in oral hard tissues is a high prevalence of caries,^25^ due to the fact that drug users commonly neglect their oral hygiene.^24^, ^26^

A significant reduction in salivary flow was observed among cocaine users, with reports of xerostomia.^19^, ^33^, ^39^ Moreover, cases of ageusia were described, and when drug addicts were compared to non-users, addicts showed a loss of taste especially regarding the sweet, bitter and salty flavors, indicating that cocaine abuse causes salivary and gustatory alterations,^24^ caused by the damage to taste receptors, either directly or through secondary processes, altered production and composition of saliva and mucosal elements, changes in the processing of sensory information related to the palate and cortex^24^ and oral mucosa dryness.^19^, ^33^, ^39^

The physical examination is the fastest and most efficient way to identify palatal lesions.^25^, ^29^, ^35^ For individual planning, two-dimensional examinations should not be the only form of diagnosis, and the use of three-dimensional images is essential, since the reference points, lines and plans facilitate a better understanding of cases with greater severity.^41^, ^42^ When necessary, to assess the extent of the lesions and have a better knowledge of their location, the use of head and neck CT can be requested,^20^, ^22^, ^23^, ^32^^,^^33^ aiming to reveal the structural relationships in depth, and show individualized images of the human body, which reduces the overlap of structures, thus obtaining a clear image.^43^

Still in this context, it is important to emphasize that a good medical history can better direct the professional towards a more assertive diagnosis and treatment.^31^, ^44^ As the lesions investigated in the present study are associated with the use of illicit substances, sometimes the user/patients can deny their use,^21^ making diagnosis difficult and delaying the start of the therapy.

In addition to the tomography, histopathological examinations of incisional biopsies of these lesions are carried out, which can often disclose the existence of acute, chronic and necrotizing inflammatory characteristics,^19^, ^32^ or even lacking necrotizing characteristics.^38^ These examinations can be extremely important in clinical diagnosis, as different types of diseases can cause destructive lesions in the oral cavity that are similar to lesions caused by cocaine use.^31^

The incidence of palatal perforations caused by cocaine use is likely to increase when left untreated.^20^ More extensive defects can directly impair functions such as speech and mastication; however, obturator prostheses can minimize these problems, as they are a conservative and non-invasive form of treatment.^20^ Another possible therapy is surgical intervention.^20^, ^21^, ^23^, ^27^^,^^34^ However, it is observed that treatment with an obturator prosthesis, as a palliative and less invasive measure, is often sufficient.^28^, ^33^, ^39^

In specific cases of destructive lesions of the facial midline caused by cocaine abuse,^23^ surgical reconstruction procedures are recommended. This surgical reconstruction can be especially indicated when the defect is located in the soft palate.^27^ In cases where the hard and soft palate tissues are affected, there is an alternative, in which these structures are removed and subsequently replaced by a prosthesis, part of which is made of acrylic for the hard palate, whereas the other part is made of a resilient and malleable material used to replace the soft palate.^29^

Another option is to use the Le Fort I osteotomy surgical technique and the use of a bilateral Bichat’s fat pad flap, which is an effective method for the correction of small and medium-sized oronasal communications. This technique is easy to perform, and has a minimal impact on the patient’s aesthetic appearance.^34^ In this sense, this procedure is an effective alternative for young and elderly patients, guaranteeing an excellent intraoral approach in addition to great aesthetic result.

The present study has limitations that must be considered, as the studies used in this review may not represent the general population,^15^, ^19^ had a small sample size,^26^, ^37^ and showed difficulties locating the users that were addicted to a single drug.^37^ In the study by Cury,^26^ a sampling error of 5% was found and no correction factor was used; men addicted to cocaine were less cooperative during the oral examination, and there was also the possibility of bias in the response to the questionnaires applied to participants.

## Conclusion

Several oral manifestations caused by cocaine abuse were identified, with the main ones being: palatal perforation, predisposition to periodontal disease, damage to oral tissues and presence of dental caries. Different approaches were used to attain a diagnosis, which had a direct effect on treatment. Therefore, it is necessary for health professionals to be able to recognize these alterations and manifestations, so that an accurate and assertive diagnosis and treatment planning can be carried out. A public health program aimed at the early diagnosis and treatment of lesions resulting from drug abuse is vital to improve the oral health of individuals who abuse cocaine.

## Conflicts of interest

The authors declare no conflicts of interest.
